# Micro-MRI Study of Cerebral Aging: Ex Vivo Detection of Hippocampal Subfield Reorganization, Microhemorrhages and Amyloid Plaques in Mouse Lemur Primates

**DOI:** 10.1371/journal.pone.0056593

**Published:** 2013-02-27

**Authors:** Anne Bertrand, Adrien Pasquier, Alexandra Petiet, Christopher Wiggins, Audrey Kraska, Nelly Joseph-Mathurin, Fabienne Aujard, Nadine Mestre-Francés, Marc Dhenain

**Affiliations:** 1 CNRS (Centre National de la Recherche Scientifique), URA2210 (Unité de Recherche Autonome 2210), Fontenay-aux-Roses, France; 2 CEA (Commissariat à l'Energie Atomique et aux Energies Alternatives), DSV (Direction des Sciences du Vivant), I2BM (Institut d'Imagerie BioMédicale), MIRCen (Molecular Imaging Reseach CENter), Fontenay-aux-Roses, France; 3 CEA (Commissariat à l'Energie Atomique et aux Energies Alternatives), I2BM (Institut d'Imagerie BioMédicale), Neurospin, Gif-sur-Yvette, France; 4 UMR CNRS/MNHN 7179 (Unité Mixte de Recherche, Centre National de la Recherche Scientifique/Muséum National d'Histoire Naturelle), Mecadev (MECanismes ADaptatifs et EVolution), Brunoy, France; 5 INSERM U710- EPHE-UM2 (Institut National de la Santé et de la Recherche Médicale, Ecole Pratique des Hautes Etudes, Université Montpellier 2), Montpellier, France; University of Cambridge, United Kingdom

## Abstract

Mouse lemurs are non-human primate models of cerebral aging and neurodegeneration. Much smaller than other primates, they recapitulate numerous features of human brain aging, including progressive cerebral atrophy and correlation between regional atrophy and cognitive impairments. Characterization of brain atrophy in mouse lemurs has been done by MRI measures of regional CSF volume and by MRI measures of regional atrophy. Here, we further characterize mouse lemur brain aging using *ex vivo* MR microscopy (31 µm in-plane resolution). First, we performed a non-biased, direct volumetric quantification of dentate gyrus and extended Ammon's horn. We show that both dentate gyrus and Ammon's horn undergo an age-related reorganization leading to a growth of the dentate gyrus and an atrophy of the Ammon's horn, even in the absence of global hippocampal atrophy. Second, on these first MR microscopic images of the mouse lemur brain, we depicted cortical and hippocampal hypointense spots. We demonstrated that their incidence increases with aging and that they correspond either to amyloid deposits or to cerebral microhemorrhages.

## Introduction

In humans, post mortem studies of brain weight or brain volume and *in vivo* magnetic resonance imaging (MRI) studies have shown that a progressive cerebral atrophy starts during the adolescence, and accelerates after the 5^th^–6^th^ decade [1]–[3]. A clear relationship has been established between age-related atrophy of specific brain regions and age-related decline in performance for the corresponding cognitive tasks: for example, hippocampal atrophy correlates with decline in memory performance [4], [5]. During neurodegenerative diseases, cerebral atrophy can be more pronounced and display a regional specificity: for example, Alzheimer's disease (AD) is associated with a fast process of atrophy predominating in the medial temporal lobe, and in the hippocampal formation in particular [6], [7].

Animal models of aging and associated neurodegenerative diseases represent a critical step for the understanding of brain aging mechanisms, and for drug development. Although transgenic mice remain the most widely used models, most of them do not reproduce the progressive cerebral atrophy observed in humans during normal aging and neurodegenerative diseases [8]. Natural models of aging and neurodegeneration, such as primates, represent an interesting alternative to transgenic animals. Mouse lemur primates (*Microcebus murinus*) are small primates: about 12 cm, 100 g, with a brain weighting less than 2 g. They can be raised in captivity with a mean and maximum life span of 5 and 12 years, respectively [9]. Aging in mouse lemurs is associated with behavioral and cognitive alterations [10], [11] and deposition of iron and lipofuscin in the brain [12]. Additionally, a subset of animals exhibits intracellular amyloid deposits and altered tau proteins, which are associated in humans with Alzheimer's disease [11], [13], [14]. During aging, a progressive cerebral atrophy appears in about 60% of aged animals [14]–[17], affecting several regions such as the caudate and splenium [18]. Some other regions such as the septum, the cingulate and the hippocampus are atrophied only in a subset of aged animals [18]. More interestingly, in lemurs, as in humans [4], [19], there is a good correlation between age-related atrophy of the hippocampus and decline in spatial memory performance [18]. To our knowledge, such a correlation between regional age-related atrophy and decline in the corresponding cognitive task has never been reported in other non-human primates [20]–[22]. Thus, mouse lemurs appear as an efficient and valuable model of brain aging.

The aim of the current article was thus to further characterize cerebral aging in mouse lemurs by using MR microscopy. First, we focused on the atrophy of hippocampus subfields. In humans, recent studies have emphasized the interest of studying hippocampal atrophy at the scale of its subfields. Indeed, the hippocampal formation comprises multiple interconnected subfields (CA1-4, dentate gyrus, fimbria, subiculum and parasubiculum), each of them differentially affected by aging, AD-related pathology, or vascular changes [23]–[25]. Recent studies have been performed in humans based on the delineation of individual subfields: subiculum, CA1, CA2, CA3, plus the region of CA4 and dentate gyrus (or, a common region grouping CA3-CA4-dentate gyrus). These studies have shown that age-related memory decline is associated with a shrinkage of CA1 and sometimes of the CA3-CA4-dentate gyrus group, while Alzheimer's disease is associated with a shrinkage of CA1, subiculum and entorhinal cortex, with a preserved volume of the CA3-CA4-dentate gyrus group [26]–[28]. In the current study, we imaged a panel of mouse lemurs aged from one to ten years using *ex vivo* MR microscopy after a passive staining protocol [29]. This imaging method allows for a 3D visualization of brain structures without slicing the tissue, and provides a spatial resolution close to histology [30]. We show an age-related reorganization of the hippocampus leading to a growth of the dentate gyrus and an atrophy of the extended Ammon's horn, even in the absence of global hippocampal atrophy. In addition to the study of hippocampal atrophy, our high-resolution images also allowed to detect hypointense spots in the cortex and hippocampal formation of lemurs. We demonstrated that their incidence increases with aging, and that they correspond either to amyloid deposits or to cerebral microhemorrhages that can both be detected by MRI.

## Materials and Methods

### 1. Materials

We studied the brains of six young (1 to 4 years of age, mean = 2.5) and six aged (6 to 10 years of age, mean = 7.6) mouse lemurs. Animals were born and raised within our breeding colonies (École Pratique des Hautes Etudes, France, license approval N°34-05-026-FS; and MNHN, France, license approval N°A91-114-1), according to the guidelines of the French Ministère de l'Agriculture (Decree 87–848), the European Community Directive (86/609/EEC), and the regional ethic committee for animal experimentation (CEEA-LR-1002). The CEEA-LR approved this specific study. The general conditions of captivity were constant. Animals were exposed to ambient room temperature (24–26°C) and relative humidity (55%). Animals were housed in groups (of less than 6 individuals) or individually depending on the period of the year and the ability of animals to interact with other partners (minimal size of the cages = 200×100×100 cm^3^ and 50×40×30 cm^3^, respectively). Animals were fed fresh fruits and a laboratory daily-made mixture of cereals, milk and egg. Water and food were given ad libitum. Environmental enrichment was provided by putting tree branches and several nests in the cages. All mouse lemurs involved in the current study died of natural causes and no mouse lemurs were sacrificed for this study. Animal brains were extracted and formalin-fixed for at least 6 months after the death of the animals. They then were stained by a one-week soaking in a solution of Gadolinium (Dotarem©, Guerbet, France) in PBS at 2.5 mmol/l. This protocol enhances the signal- and contrast-to-noise ratios on MR images of fixed brains [29], and facilitates the detection of cerebral amyloid deposits as shown in transgenic mice both *ex vivo* and *in vivo* [29], [31].

### 2. MR image acquisition

MR images were recorded on a 7T clinical magnet (Siemens, Syngo MR VB15), with gradients' strength of 80 mT/m and a slew rate of 333 mT/m/s, using a surface coil with an inner diameter of 2.5 cm. A 3D gradient echo T_2_*-weighted sequence was used (TE = 20.8 ms; TR = 200 ms; flip angle = 80°, bandwidth = 50 Hz/pixel), with a field-of-view of 24×20.3 mm, a matrix of 768×648 and a slice thickness of 120 µm, leading to a spatial resolution of (31×31×120) µm^3^. Imaging time was comprised between 5 and 6 hours.

### 3. Segmentation of the hippocampus

**Figure 1:**
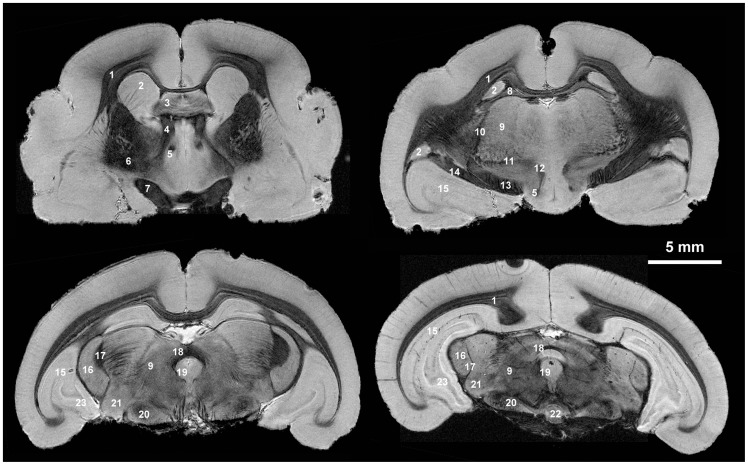
resolution MR images of the mouse lemur brain at 7T. 1: corpus callosum; 2: caudate nucleus; 3: septal nuclei; 4: thalamic medullary stria; 5: fornix; 6: lenticular nucleus; 7: optic chiasm; 8: hippocampal fimbria; 9: thalamus; 10: external medullary lamina; 11: Forel's field; 12: mamillo-thalamic tract; 13: cerebral peduncle; 14: optic tract; 15: hippocampus; 16: lateral geniculate body; 17: median lemniscus; 18: posterior commissure; 19: central grey matter; 20: substantia nigra; 21: medial geniculate nucleus; 22: central interpedoncular nucleus; 23: dentate gyrus. Based on [32].

**Figure 2:**
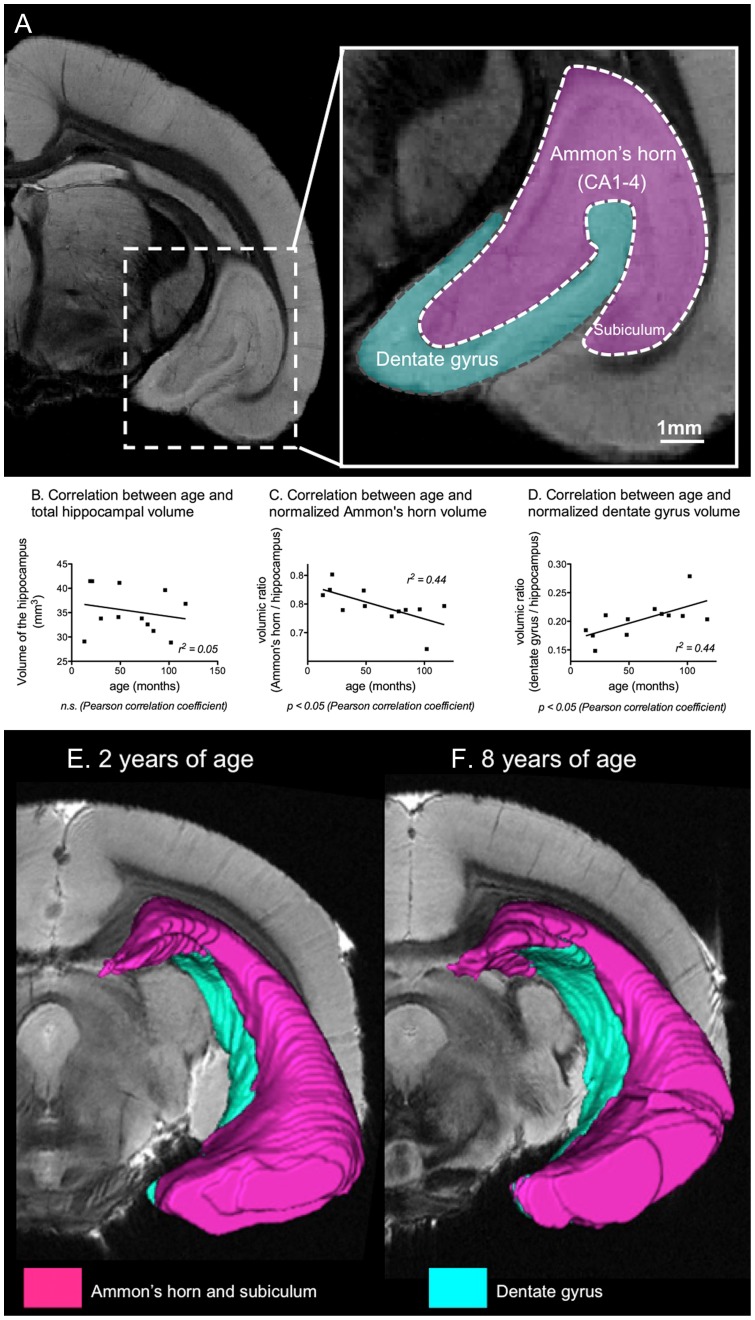
Analysis of hippocampal subfield volumes. (A) Segmentation of the hippocampus, divided into the dentate gyrus and the extended Ammon's horn (includes CA1, CA2, CA3, CA4 and subiculum), based on the mouse lemur brain atlas [48]. No correlation was found between age and total hippocampal volume (B), however, we observed a significant decrease with age of the normalized volume of the extended Ammon's horn (C), and a significant increase with age of the normalized volume of the dentate gyrus (D). (E–F) 3D views of hippocampal subfields in one young (E) and one old animal (F), showing the increased volume of the dentate gyrus and decreased volume of the Ammon's horn in the aged lemur as compared to the young one.

MR images were analyzed using Anatomist freeware (Anatomist 3.1.6, http://brainvisa.info/index_f.html). Segmentation was performed on the right hemisphere. We manually delineated the dentate gyrus and the extended Ammon's horn (CA1-4 and subiculum) (Fig. 1,2). We calculated their respective volumes, and we normalized both volumes to the total hippocampal volume. We could not segment individually the subfields CA1 to CA4 and the subiculum, because their respective pyramidal cell layers are continuous, and their histological boundaries have not been assessed so far in mouse lemurs [32]. Thus, the whole segmentation process performed in our work relied on the direct visualization of individual cell layers.

### 4. Identifications of cerebral dark spots

Dark spots visible in the cortex and hippocampus were manually counted in the brain of each animal. Dark spots were defined as tiny (less than 10 pixels), round hypointense areas, and could not be followed over more than 2 adjacent slices, thus they did not correspond to a section of vessel.

### 5. Histology

Right hemispheres were used for histology. After cryoprotection in 15%, then 30% sucrose solution, brains were frozen and sliced into 40-µm-thick coronal sections on a freezing microtome. Slices were then stored at −20°C in a storage solution. Adjacent sections were stained for amyloid-β (immunohistochemistry) and iron (Perls and Perls-DAB coloration) as previously described [33]. For Aβ staining, we used a mouse monoclonal 4G8 antibody diluted at 1/1000e (SIGNET, USA), revealed with an avidin–biotin–peroxidase method (Vectastain, VECTOR, USA); negative controls were performed by omitting the primary antibody, and positive controls by using brain samples of patients with Alzheimer's disease. All slices were digitized using a Super CoolScan 8000 ED high-resolution scanner (Nikon, France).

### 6. Statistical analysis

Data were analyzed using GraphPad Prism 5.0 for Mac OSX (San Diego, CA). Correlations between age, hippocampal volumes and number of dark spots were assessed by Pearson correlation coefficient, except when the data failed at least two of the three normality tests (D'Agostino and Pearson, Shapiro–Wilk and Kolmogorov-Smirnov normality tests) or the test of equal variances. In these cases correlations were assessed using the non-parametric Spearman correlation coefficient.

## Results

We implemented a new protocol to record 3D MR microscopic images ([31×31×120] µm^3^) on a clinical spectrometer. The recorded images showed a high contrast between hypointense white matter and hyperintense gray matter. They allowed a fine discrimination of small fiber bundles such as the fornix, the mamillo-thalamic tract, the thalamic medullary stria and the hippocampal fimbria. Tiny perforating white matter tracts could be individualized in the caudate nucleus or in the vicinity of the median lemniscus. Individual cell layers could be clearly distinguished within the hippocampal formation (Fig. 1).

On these high-resolution images, direct visualization of dentate gyrus granular layer as well as Ammon's horn and subiculum pyramidal layer, allowed us to measure their respective volumes (Fig. 2A). The normalized volume of extended Ammon's horn was negatively correlated with the age of the animals (r^2^ = 0.44, p<0.05, Fig. 2C). The normalized volume of the dentate gyrus was positively correlated with the age of the animals (r^2^ = 0.44, p<0.05, Fig. 2D). This latter variation was already present as a trend when the dentate gyrus volume was not normalized (p = 0.06, data not shown). The total hippocampal volume was not significantly correlated with the age of the animals (n.s., Fig. 2B). Normalized volumes of the extended Ammon's horn and dentate gyrus were highly correlated (p<0.0001, Pearson correlation coefficient, data not shown).

**Figure 3:**
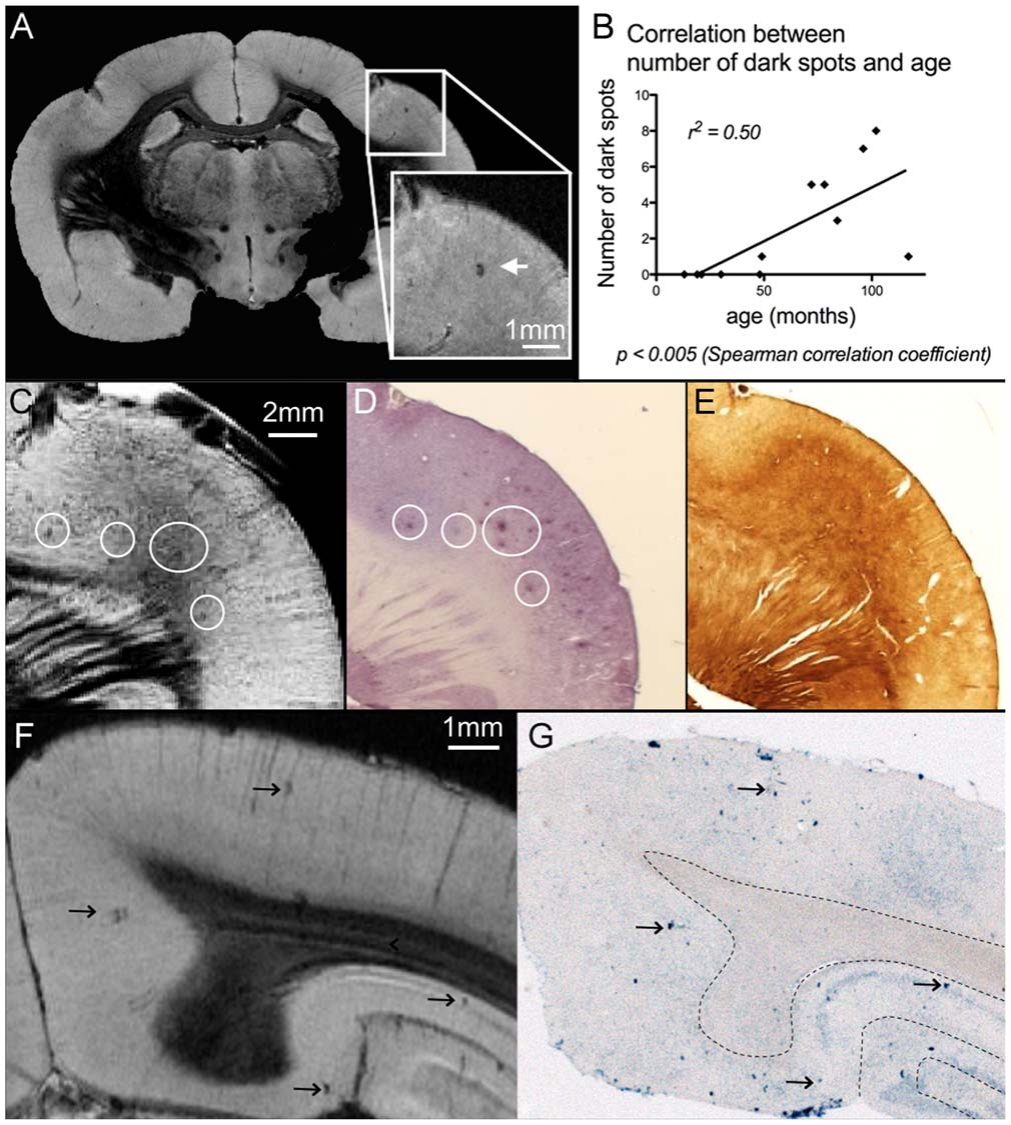
Detection of cortical and hippocampal black dots. (A) Example of the black dots detected in the cortex of mouse lemurs on MR images. (B) The number of black dots in each animal was significantly correlated with age (p<0.005). In one animal, some black dots visible on MR images (C) matched the location of amyloid deposits detected by Abeta staining (D, 4G8 staining, white circles). The level of iron within amyloid deposits was similar or slightly higher than in the surrounding parenchyma (E, Perls-DAB staining). In animals without amyloid deposition, the location of black spots on MR images (F, arrows) corresponded to focal area of microhemorrhages detected by Perls staining (G, arrows).

Our high-resolution images allowed detecting small dark spots (Fig. 3A) in the cortex and hippocampus of all aged animals; a dark spot was also present in the cortex of a 4 year-old lemur. The number of dark spots in each animal was significantly correlated with the age of the animals (r^2^ = 0.5, p<0.005, Fig. 3B) and with the normalized volume of extended Ammon's horn and dentate gyrus (r^2^ = 0.5 p<0.005, data not shown). Only one animal displayed extracellular amyloid deposits, although its amyloid load was low. In this aged animal, we were able to co-register some of the hypointense spots detected by MRI with extracellular amyloid deposits (Fig. 3C–D). The level of iron in these amyloid deposits was similar to its level in the surrounding parenchyma (Fig. 3E, Perls-DAB staining). In the other amyloid deposit free animals, some dark spots could be registered with microhemorrhages detected on Perls stained sections (Fig. 3F–G), although not all microhemorrhages detected on Perls stained sections could be registered with dark spots on MR images. Noticeably, none of the animals had evidence of amyloid angiopathy, even in the cases where microhemorrhages were present on Perls staining.

## Discussion

### 1. Age-associated reorganization of hippocampal subfields

In this work, we recorded *ex vivo* MR microscopic images of mouse lemur brains and used them to evaluate the volumes of hippocampal subregions. *Ex vivo* MR imaging allows for 3D visualization of brain structures without slicing the tissue and provides a spatial resolution close to histology [30]. This allows estimating the volume of small brain structures with a better accuracy than measures in sliced brain sections. Interestingly, previous studies of hippocampal subfield in human hippocampus have shown that volumes measured on *ex vivo* MRI strongly correlate with the number of neurons in the corresponding subfields [34]. This demonstrates the interest of 3D *ex vivo* MRI as an estimate of neuronal loss. However, volumes measured on *ex vivo* MRI are probably smaller than real volumes that would be measured by *in vivo* MRI. Indeed long-term formalin fixation is known to induce a reduction in brain weight and volume [35], which depends on tissue structure and cellularity [36]. Both dentate gyrus and Ammon's horn consist of a 3-layered cortex of similar origin (*i.e.* the archipallium) [37], and thus their respective volumes are expected to be similarly affected by the fixation process. Also, all brains used in our study were fixed for more than 6 months: because of this long period, fixation-induced changes are expected to be stabilized [35]. Still, the fixation process may cause underestimation of an age-related atrophy of the hippocampus, because the shrinkage caused by long-term formalin fixation is more pronounced in juvenile animals as compared to other animals (as shown in dog [38], rat and mouse brains [36]).

We showed an age-associated rearrangement of hippocampal subfields in mouse lemurs, with a decrease of extended Ammon's horn relative volume and an increase of dentate gyrus relative volume in aged animals. We did not detect any significant modification of the total hippocampal volume during aging. However, it has been shown *in vivo* that hippocampal atrophy does not occurs in all aged mouse lemurs but only in a subset, and thus reflects more a pathological process than normal aging [18]. In our study group, we did not observe severe amyloid lesions: sparse amyloid deposits were detected in only one aged animal, in coherence with the reported 5–10% incidence of amyloid deposits in aged mouse lemurs [39]. Hence, our work gives a picture of normal aging in mouse lemurs, rather than of Alzheimer's like changes, and the lack of detected hippocampal atrophy is in coherence with *in vivo* observations showing a preserved hippocampal volume in most of the aged animals [18].

The age-associated growth of the dentate gyrus that compensated the atrophy of the extended Ammon's horn was an unexpected result. These two areas are part of the hippocampal circuitry, and are directly connected via the mossy fibers. Although both of them are affected during normal aging, they present distinct functional changes: in rats, a loss of synapses is observed in the dentate gyrus during aging. In CA1, the number of synapses is preserved but they are functionally silent [40]. Interestingly, the dentate gyrus is the site of adult hippocampal neurogenesis, as shown in both rodents [41] and primates [42]. Thus, the dentate gyrus might be activated to compensate for hippocampal cell loss [42], [43]; this could explain the opposite changes in volume observed in these two regions in aged animals.

To our knowledge, our work is the first report of a direct MR volumetry of the dentate gyrus. We showed that this measurement is feasible in non-human primates and can give new informations on microscopical signatures of aging. The study of hippocampal subregions is also a new field of investigation in humans, and has been fostered by the development of ultra-high field clinical systems [26], [27], [44]. However, the spatial resolution of *in vivo* MRI (above 400 microns) does not allow visualizing dentate gyrus landmarks, and human studies have always evaluated the volume of the dentate gyrus grouped with those of the CA3 and CA4 subfields. This is questionable, as dentate gyrus on the one hand and Ammon's horn on the other hand show very different cellular structures [37]. In humans a shrinkage of the CA3-CA4-dentate gyrus group during normal aging has been reported in one *in vivo* study [27] but was not found in another one [45]. More specific volumetric studies in humans using *ex vivo* MRI would be of interest, in order to specifically delineate the dentate gyrus. Such studies would help understanding whether the observed shrinkage of CA1 and CA3-CA4-dentate gyrus during aging is related to an atrophy process limited to the Ammon's horn (from CA1 to CA4), or to a process affecting both Ammon's horn and dentate gyrus.

### 2. Age-associated occurrence of microhemorrhages

In the most aged mouse lemurs, several dark spots depicted on MR images could be registered with focal microhemorrhages. One can however note that microhemorrhages depicted on Perls were more numerous that the black dots seen on MR images. This can be related to the inability of MRI to detect very small microhemorrhages that can be detected on histological sections [46]. Different thickness of histologic sections (40 µm) and MRI slices (120 µm), leading to partial volume effects on MR images, could also explain the imperfect matching between spot detected on MRI and microhemorrhages seen on histology.

To our knowledge, cerebral microhemorrhages have not been reported so far in mouse lemurs. In humans, *ex vivo* studies have shown that cortical microhemorrhages are a common feature of the aging cerebral cortex [47]. Clinical detection of deep microhemorrhages in patients has been suggested to be a marker of microvascular disease [48]. Our data suggest that, as in humans, vascular alterations are frequent in old lemurs, and are part of the brain aging process. We however did not observe any evidence of large hemorrhages or ischemic strokes in the brain of the animals. In the present study, the number of dark spots was significantly correlated to the normalized volume of extended Ammon's horn (data not shown). However, because both findings were correlated with the age of the animals, we cannot conclude if microhemorrhages and extended Ammon's horn atrophy reflect two independent or joined processes during brain aging.

### 3. Detection of amyloid plaques by MRI

In one aged animal, we were able to detect amyloid deposits on MR images. MRI detection of amyloid deposits is well established in transgenic mouse models [49], [50] but remains highly debated in humans [51], [52]. This discrepancy may be related to the different microscopic structure of amyloid deposits between transgenic mice and humans: in transgenic mice, amyloid deposits consist of highly-packed amyloid fibril aggregates with low levels of iron, whereas in patients with sporadic Alzheimer's disease, it consists of smaller aggregates of less-packed fibrils with higher levels of iron [53]. Hence, because of these intrinsic differences, the possibility to detect amyloid plaques by MRI in transgenic mice does not necessarily mean that this can also be achieved in humans. Our work demonstrate that mouse lemur amyloid deposits, consisting of diffuse deposits with low levels of iron, can still be detected on MR images: their microscopic structure do not jeopardize their detection by MRI. This suggests that amyloid detection by MRI is physically possible in primates, and thus probably in humans. In our protocol, we used a passive staining method, which enhances the contrast between amyloid deposits and the surrounding brain tissue. This method has been validated in transgenic mouse models initially *ex vivo* [29], and then *in vivo*, using intraventricular injections of a Gadolinium chelate [31]. Hence, our work extends this method to the field of non-human primates, and represents the ultimate step before its use *in vivo* in non-human primates. This would represent a useful tool for the non-invasive, longitudinal evaluation of amyloid load during therapeutic trials of anti-amyloid treatments [54].

## Conclusion

To conclude, our micro-MRI study highlights new morphological MR signature of brain aging in mouse lemurs. First, we showed a reorganization of hippocampal subfields leading to a growth of the dentate gyrus. Second, we showed that microhemorrhages occur during aging in mouse lemurs. Vascular alterations thus seem to be part of the age-related cerebral changes observed in this primate. Third, we showed that amyloid plaques can be detected in the brain of this primate by MRI with the passive staining method. As the plaques are developed in a natural way in this primate, they are expected to be closely comparable to those developed in humans. This study thus reinforces the concept that, in the future, MRI can be a tool for the detection of amyloid plaques in humans.
